# Antiproliferative Effect of 24‐Deoxysericoside From *Terminalia macroptera* Guill. & Perr. (Combretaceae) Against Breast Carcinoma: In Vitro, Molecular Docking and ADME Assessment

**DOI:** 10.1002/cnr2.70541

**Published:** 2026-04-07

**Authors:** Romeo Toko Feunaing, Alfred Ngenge Tamfu, Abel Joel Yaya Gbaweng, Cyrille Leonel Tchuente Djoko, Emmanuel Talla, El Hassane Anouar, Stephane Zingue

**Affiliations:** ^1^ Department of Chemistry, Faculty of Sciences University of Ngaoundere Ngaoundere Cameroon; ^2^ Department of Chemical Engineering, School of Chemical Engineering and Mineral Industries University of Ngaoundere Ngaoundere Cameroon; ^3^ Department of Materials Engineering, School of Chemical Engineering and Mineral Industries University of Ngaoundere Ngaoundere Cameroon; ^4^ Department of Chemistry, College of Sciences and Humanities in Al‐Kharj Prince Sattam bin Abdulaziz University Al‐Kharj Saudi Arabia; ^5^ Department of Pharmacotoxicology and Pharmacokinetics, Faculty of Medicine and Biomedical Sciences University of Yaounde 1 Yaounde Cameroon

**Keywords:** breast cancer, deoxysericoside, molecular docking, *Terminalia macroptera*, triterpenoid saponins

## Abstract

**Background:**

*Terminalia macroptera* (Combretaceae) is an important medicinal plant in the traditional pharmacopeia in most tropical areas, where its different parts are used in treating illnesses including cancer.

**Aims:**

In this study, two oleanane‐type triterpenoids: terminolic acid (TM32) and arjungenin (TM34), together with three saponins: arjunglucoside I (TM35), 24‐deoxysericoside (TM36) and chebuloside II (TM37) from *T. macroptera*, were screened for their cytotoxic effects against breast cancer cell lines.

**Methods and Results:**

The compounds were isolated using column chromatography and characterized from their NMR data. Their cytotoxic and antiproliferative effects against estrogen non‐sensitive (MDA‐MB 231) and estrogen sensitive (MCF‐7) breast cancer cell lines were evaluated. Against estrogen non‐sensitive (MDA‐MB 231) cancer cell lines, deoxysericoside (TM36) was profoundly active compared to the control. Terminolic acid (TM32), arjungenin (TM34), and arjunglucoside I (TM35) were also active. Against estrogen sensitive (MCF‐7) breast cancer cell lines, deoxysericoside (TM36) exhibited significant activity (*p* < 0.05) compared to control experiments. The most active compound had an optimum concentration of 30 μg/mL. Deoxysericoside (TM36) showed concentration‐dependent inhibition percentages at 15 and 30 μg/mL, and MDA‐MB 231 breast carcinoma cells were more susceptible. MDA‐MB 231 treated with 15 and 30 μg/mL of deoxysericoside (TM36) showed significant reduction (*p* < 0.05) in clone formation after 48 h when compared to untreated controls, suggesting that it can restrict cancer to a preliminary stage. The deoxysericoside (TM36) reduced cell migration with dose‐dependent improvement in wound healing at 15 and 30 μg/mL, revealed by the micrographs. Molecular docking indicated that the compounds fit well into hERα and PI3Kα receptor binding sites, forming stable complexes with binding energies in ranges of −9.04 to −5.02 kcal mol^−1^ (hERα receptor) and −8.84 to −5.97 kcal mol^−1^ (PI3Kα receptor). The compounds exhibited appreciable drug likeness predicted using SwissADME.

**Conclusion:**

The studies showed that the isolated compounds could be used for the development of anticancer therapies.

## 1 Introduction

Cancer is a multifactorial genetic disease causing uncontrolled division and proliferation of abnormal cells which can spread across tissues [1]. It ranks as one of the leading causes of death and an important barrier to increasing life expectancy in every country of the world. It represents a public health problem and is the second leading cause of death worldwide, responsible for more than 10.0 million deaths out of 19.3 million cases recorded in 2020 [2]. The global cancer burden is expected to be 28.4 million cases in 2040, a 47% rise from 2020, with a larger increase in developing countries (64%–95%) versus developed countries (32%–56%) according to demographic changes [3]. Among these cancers, breast cancer (BC) is one of the deadly diseases that affects female lives globally [4, 5]. According to the World Health Organization (WHO), the number of women diagnosed with breast cancer worldwide is approximately 2.3 million, while 685 000 died from the disease [4]. As a result, breast cancer is the most prevalent type of cancer, the second leading cause of woman cancer‐related deaths globally, and the fifth most common cause of cancer related deaths overall [4, 6]. Treatment for breast cancer involves hormonal, radiation, and chemotherapy therapies. The outcomes are frequently influenced by the subtypes which include hormone receptor positive, human epidermal growth factor receptor 2 (HER2) amplified and triple‐negative tumor cells [7].

To overcome this disease, several treatments are used such as surgery, radiation therapy, cryotherapy, chemical ablation, chemotherapy, hormonal therapy, immune therapy, and targeted therapy [8]. Medicinal plants and their constituents are also used as alternatives to chemotherapeutic treatments for breast cancers as they cause minimal side effects when compared to chemo‐drugs [9, 10]. The increase in morbidity and mortality from cancer in recent years has led to an increased number of studies on chemotherapies and the identification of new anti‐cancer agents aimed at obtaining more effective drugs [11]. Research for new natural anti‐cancer agents is an important area for the improvement of cancer therapy [12]. There is recent growing interest in alternative and complementary medicine for cancer patients. More than 60% of anticancer drugs originate from natural sources, mostly medicinal plants selected on the basis of their phytochemical composition and ethnopharmacological properties [13, 14]. Terpenoids and isoprenoids are among the potential anticancer drug leads obtained from natural sources and they are one of the most abundant secondary metabolites in plants [4]. They are classified into different categories based on the number of isoprene units, such as monoterpenes, sesquiterpenes, diterpenes, sesterterpenes, triterpenes, tetraterpenes, and polyterpenes [15, 16, 17]. Triterpenoids, a subclass of terpenoids, have lately emerged as a unique category of phytochemicals with multifunctional anticancer properties, as evidenced by preclinical results [4, 18]. Saponins are glycosides with steroid, triterpenoid or spirostane aglycones and they have shown pharmacological effects including anticancer properties, thereby gaining attention in cancer chemotherapy [19, 20, 21, 22]. Saponins exert anticancer properties via various mechanisms being able to regulate angiogenesis, cytotoxicity, apoptosis and inflammation in various human cancer cell lines [23].

There is growing interest in the anticancer and chemopreventive properties of triterpenoids and their corresponding saponins. In the present work, oleanane‐type triterpenoids and their saponins isolated from *Terminalia macroptera* were evaluated for their cytotoxic potential on estrogen‐sensitive MCF‐7 and non‐sensitive MDA‐MB 231 breast cancer cell lines. Molecular docking and prediction of pharmacokinetic properties of the compounds were studied. The most potent compound was further investigated for its antiproliferative effects.

## 2 Materials and Methods

### 2.1 Extraction, Isolation, and Characterization of Compounds

Extraction, isolation, and characterization of compounds (TM32, TM34, TM35, TM36, and TM37) from the plant *Terminalia macroptera* were performed as described previously [24].

### 2.2 Antiproliferative Activity

#### 2.2.1 Substances & Reagents

From Gibco/Invitrogen (Karlsruhe, Germany) were purchased penicillin, fetal bovine serum (FBS), and streptomycin; Roche Diagnostics (Penzberg, Germany) supplied the 3‐(4,5‐dimethylthiazol‐2‐yl)‐2,5‐diphenyltetrazolium bromide (MTT) dye reducing assay kit; and Becton Dickinson (BD) Pharmingen (Heidelberg, Germany) supplied the Annexin V‐FITC Apoptosis detection and cycle TEST PLUS DNA reagent kits.

#### 2.2.2 Cell Lines and Cell Cultures

Human estrogen‐sensitive (MCF‐7) and estrogen‐non‐sensitive (MDA‐MB 231) cell lines were provided by Promochem American Type Culture Collection (ATC/LGC) in Wesel, Germany. RPMI‐1640 medium supplemented with 10% fetal bovine serum (FBS) and 1% penicillin (100 U/mL)/streptomycin (100 g/mL) was used to cultivate and subculture MCF‐7 and MDA‐MB 231 cells. A humid 5% CO_2_ incubator kept at 37°C and pH 7.4 was used to incubate them. Fresh medium was added every two days to replace 90% of the supernatant for cell passage. Using the Multiskan TECAN reading counter system and the enzyme‐linked immunosorbent assay (ELISA), the number of viable cells was calculated before each experiment.

#### 2.2.3 Screening of Compounds for Cytotoxic Activity

The MTT dye reduction test (Roche Diagnostics, Penzberg, Germany) was used to measure cell proliferation. MDA‐MB 231 cells (100 μL, 1 × 10^5^ cells/mL) and treated versus untreated MCF‐7 cells were sown onto 96‐well plates. In order to screen for potential cytotoxicity, the compounds TM32, TM34, TM35, TM36, and TM37 were dissolved in dimethyl sulfoxide (DMSO) and evaluated at final concentrations of 15, 30, and 60 μg/mL. In order to determine its effect on cell proliferation after 24, 48, and 72 h, the most active compound in terms of cytotoxicity, considered as the most promising (TM36), was further examined at concentrations ranging from 12.5 to 200 μg/mL. Only the vehicle (DMSO 0.01%) was exposed to the controls. After adding 10 μL of MTT (5 mg/mL) at 0, 24, 48, and 72 h, the cells were incubated for 2 h at 37°C with 5% CO_2_. They were then lysed for two more hours in a buffer that contained 10% SDS in 0.01 M HCl. Each well's absorbance at 550 nm was measured with a microplate ELISA TECAN SPARK reader (Crailsheim, Germany).

#### 2.2.4 Assessment of Cell Proliferation

The stable and non‐toxic GLPBio Cell Counting Kit‐8 (CCK‐8) (Hamburg, Germany) was used to assess the capacity of TM36 to suppress cell proliferation. In 96‐well plates, MCF‐7 and MDA‐MB 231 cells (100 μL, 1 × 10^4^ cells/mL) with treatment and no treatment were seeded and incubated for 24 h at 37°C with 5% CO_2_. The control was then exposed to the vehicle (DMSO 0.01%). The 10 μL of TM36 at 10 to 20 μg/mL concentrations was tested. After 48 h of incubation, 10 μL of Cell Counting Kit‐8 (CCK8) solution was added to the wells and further incubated at 37°C with 5% CO_2_ for 4 h. The plates were gently homogenized on a shaker and absorbance measured at 450 nm.

#### 2.2.5 Assessment of the Inhibition of Colony Formation

MDA‐MB 231 cells treated as well as untreated ones at a density of 500 cells per well were moved into 6‐well plates. After that, TM36 was added at the ideal concentration. Colonies with at least 50 cells were counted after 7 days of incubation. The number of clones of treated tumor cells was compared to the number of clones in the control, which was set at 100%.

#### 2.2.6 Wound Healing Assay

The inhibition of MDA‐MB 231 cell migration in the presence of TM36 is evaluated. 5 × 10^5^ cells per well were seeded in 6‐well plates, and they were allowed to develop until they reached confluency. Additionally, a 100 μL pipette tip was used to generate a scratch wound, which was subsequently cleaned twice with phosphate‐buffered saline (PBS) to remove mechanically detached cells. A serum‐free RPMI 1640 medium was substituted for the original medium 4 h prior to the formation of the wound. After adding E8 or DMSO as a control solvent to serum‐free RPMI 1640 medium, cells were left for 72 h. A fluorescent microscope (10×) Olympus CK2/ULWCCD 0.030 (Olympus, Japan) was used to record the variation in the healing of the injured area by migrating cells. After every 24 h, microphotographs and wound healing areas were evaluated using ImageJ software.

### 2.3 Molecular Docking Study

Further to the experimental investigation of the cytotoxic effects against breast cancer cell lines of terminolic acid (TM32), arjungenin (TM34), arjunglucoside I (TM35), 24‐deoxysericoside (TM36), and chebuloside II (TM37), their binding affinities into the binding sites of (i) the human estrogen receptor alpha (hERα) plays a role in cell proliferation and has been found in breast cancer, and (ii) phosphatidylinositol‐3‐kinase α (PI3Kα), a therapeutic target of high interest in anticancer drug research, have been determined using the molecular docking Autodock package [25, 26]. HER2+ breast cancer protein was considered. 4JPS (phosphoinositide 3‐kinase alpha, PI3Kα) and 3ERT (human estrogen receptor alpha, hERα) were used in molecular docking studies because of their well‐characterized binding locations, relevance for biological activity, and high‐quality structural data. The hERα and PI3Kα targets with their original ligands were downloaded from the RCSB database using PDB files 3ERT and 4JPS, respectively [27, 28]. The validity of the molecular docking is investigated by re‐docking the original ligands into the binding site of hERα and PI3Kα targets, which yield RMSD values and binding energies of 0.70 Å and −14.41 kcal mol^−1^, and 0.70 Å and −14.41 kcal mol^−1^, respectively. Further details on molecular docking steps may be found in our previously reported study [24]. The 3D and 2D binding interactions of the compounds and the reference drug doxorubicin into the binding site of human estrogen receptor‐α (hERα) and phosphatidylinositol 3‐kinase (PI3K) are presented in Figures S1 and S2, respectively.

### 2.4 ADME and Druglikeness Properties

The predictions of ADME (absorption, distribution, metabolism, and excretion), druglikeness, pharmacokinetics, and physico‐chemical properties of terminolic acid (TM32), arjungenin (TM34), arjunglucoside I (TM35), 24‐deoxysericoside (TM36), chebuloside II (TM37), and doxorubicin were carried out using the Swiss ADME tool available at the https://www.swissadme.ch/ website. The obtained data are presented in Tables S1–S6. Further, the Swiss target prediction tool (https://www.swisstargetprediction.ch/) was employed to predict the probable targets of terminolic acid (TM32), arjungenin (TM34), arjunglucoside I (TM35), 24‐deoxysericoside (TM36), chebuloside II (TM37), and doxorubicin.

### 2.5 Statistical Analysis

Data analysis was performed with GraphPad Prism version 5.00 software and experiments were done in triplicate. Dunnett's post hoc test was used for comparison of data from groups and analyzed using ANOVA. For *p* value < 0.05, it was considered statistically significant.

## 3 Results

### 3.1 Isolated Compounds

**FIGURE 1:**
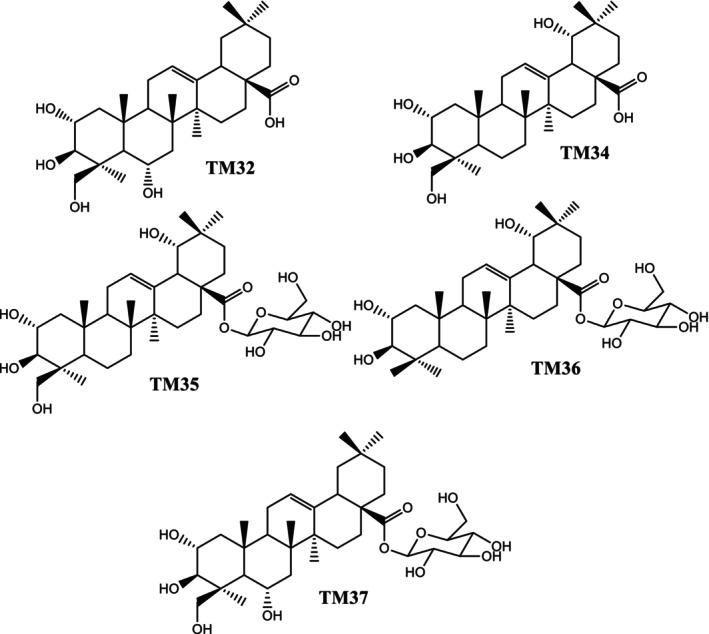
Structures of isolated compounds.

The NMR data and spectra (^1^H and ^13^C NMR) of the isolated compounds are provided in Figures S3–S7. The structures of the isolated compounds are provided on Figure 1.

### 3.2 Screening of Isolated Compounds for Their Potential Cytotoxic Effect Against Breast Cancer Cells

**FIGURE 2:**
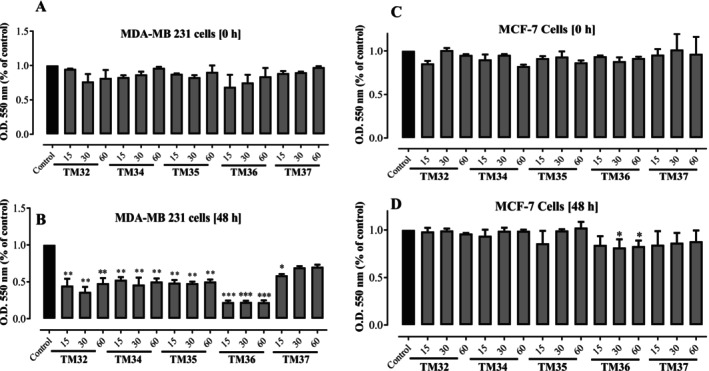
Screening of isolated compounds for their potential cytotoxic effect. MDA‐MB 231 (A & B) and MCF‐7 (C & D) tumor cells were treated for 48 h with compounds at concentrations of 15, 30, and 60 μg/mL and MTT assay was performed at 0 h and 48 h. *p < 0.05, **p < 0.01, ***p < 0.001 compared to control.

Five compounds, terminolic acid (TM32), arjungenin (TM34), arjunglucoside I (TM35), 24‐deoxysericoside (TM36), and chebuloside II (TM37) were screened at 15, 30, and 60 μg/mL for 0 and 48 h for their growth inhibitory potential against estrogen non‐sensitive (MDA‐MB 231) and estrogen sensitive (MCF‐7) breast cancer cell lines. The results are presented on Figure 2. At 0 h, it was observed that the inhibitory effects against MDA‐MB 231 were dose‐dependent for compounds arjungenin (TM34), deoxysericoside (TM36), and chebuloside II (TM37), while for terminolic acid (TM32) and arjunglucoside I (TM35), the optimal concentration was 30 μg/mL. Against MCF‐7 cell lines, the optimum concentration was 60 μg/mL for arjungenin (TM34), arjunglucoside I (TM35), 24‐deoxysericoside (TM36), and chebuloside II (TM37) and 15 μg/mL for terminolic acid (TM32). After 48 h, the inhibitory effects were more pronounced for all compounds, as shown on Figure 2. Against estrogen non‐sensitive (MDA‐MB 231) cancer cell lines, compound deoxysericoside (TM36) was profoundly active (*p* < 0.01) compared to the control. The compounds terminolic acid (TM32), arjungenin (TM34), and arjunglucoside I (TM35) were also active (*p* < 0.01) compared to the control. Against estrogen sensitive (MCF‐7) breast cancer cell lines, deoxysericoside (TM36) was the most active compound (*p* < 0.05) compared to the control experiments. From these screening results, the most active compound deoxysericoside (TM36) will be considered for further evaluations of its antiproliferative effects against both breast cancer cell lines.

### 3.3 Cell Growth/Proliferation Inhibition by Deoxysericoside (TM36)

**FIGURE 3:**
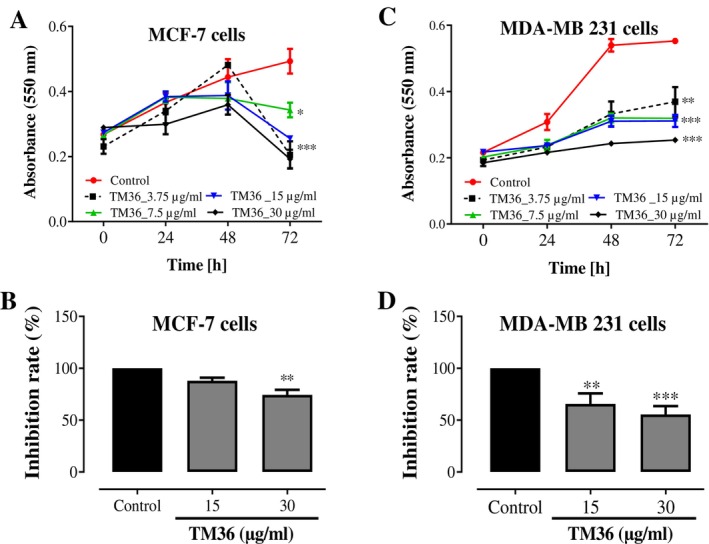
Growth and proliferation of estrogen‐sensitive MCF‐7 (A) and non‐sensitive MDA‐MB 231 (B) breast carcinoma cells treated as well as with different concentrations of compound TM36 after 24, 48 and 72 h. Controls remained untreated. (*n* = 3). Treated cancer cell cultures were compared to non‐treated control cultures of the same passage and cell numbers per well. *p < 0.05, **p < 0.01 and ***p < 0.001 compared to control.

The effect of deoxysericoside (TM36) in inhibiting the growth and proliferation of estrogen‐sensitive MCF‐7 and non‐sensitive MDA‐MB 231 breast carcinoma cells, evaluated at four concentrations (30, 15, 7.5 and 3.75 μg/mL) after 24, 48 and 72 h, are presented on Figure 3. For the MCF‐7 cell lines, from the absorbance, the inhibitions were best for the highest test concentration after 24, 48 and 72 h. The best inhibitions were observed after 72 h for all doses compared to the untreated control. The inhibition percentages against MCF‐7 carcinoma cells evaluated at 15 and 30 μg/mL indicated a dose‐dependent variation, with about 75% of cell viabilities (*p* < 0.01) at 30 μg/mL compared to untreated controls. Against the MDA‐MB 231 breast carcinoma cells, the activity decreased with time for all doses (30, 15, 7.5 and 3.75 μg/mL), but activities were good compared to untreated control. The compound deoxysericoside (TM36) exhibited good percentages of inhibition at 15 μg/mL (*p* < 0.01) and 30 μg/mL (*p* < 0.001) compared to untreated controls. Less than 50% of MDA‐MB 231 breast carcinoma cells were viable when treated with 30 μg/mL of deoxysericoside (TM36). The estrogen non‐sensitive MDA‐MB 231 breast carcinoma cells were more susceptible in the cytotoxicity and antiproliferative assays and were further considered for clone formation and cell migration inhibitory effects.

### 3.4 Inhibitory Effect of Deoxysericoside (TM36) on Clone Formation

**FIGURE 4:**
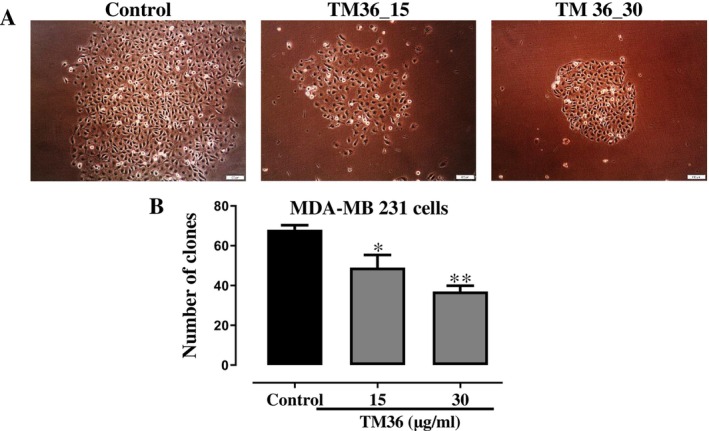
Clonogenic growth of MDA‐MB 231 cells exposed to compound TM36 at 10 and 20 μg/mL after 48 h: Graphic representation (A) and photomicrographs (B). Control remained untreated. *p < 0.05 compared to control.

The ability of deoxysericoside (TM36) to inhibit clone formation by estrogen non‐sensitive MDA‐MB 231 (B) breast carcinoma cells was evaluated and presented on Figure 4. Clonality suggests that cancer starts with a single cell and can spread to thousands or even millions of cells. Therefore inhibiting clone formation could restrict cancer to a preliminary stage. Upon exposure of MDA‐MB 231 cells to 15 and 30 μg/mL of deoxysericoside (TM36), there is an observed reduction in clone formation at after 48 h (*p* < 0.05) when compared to untreated controls as shown on Figure 4. The number of clones measured at 15 and 30 μg/mL and presented on Figure 4. The untreated control (70 clones) had more clones when compared to treatment with 15 μg/mL (50 clones) and 30 μg/mL (35 clones) of deoxysericoside (TM36).

### 3.5 Effects Deoxysericoside (TM36) on MDA‐MB 231 Cell Migration

**FIGURE 5:**
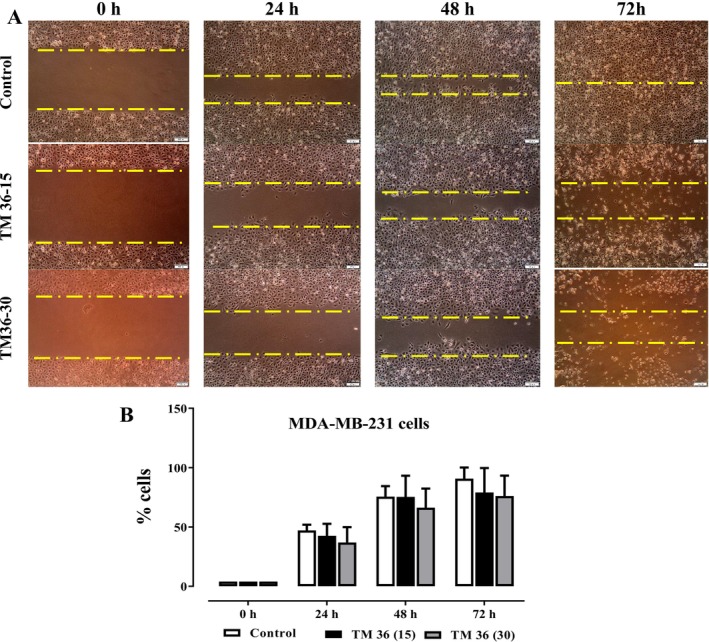
Effects of TM36 on MDA‐MB 231 cells migration. Microphotographs of one assay (A) and graphic representation of three independent wound‐healing assays (B) in MDA‐MB 231 cells migration after 72 h of treatment in serum free RPMI 1640 medium. *p < 0.05 as compared with control.

The inhibitory effects of deoxysericoside (TM36) on the cellular migration of MDA‐MB 231 breast carcinoma cells were evaluated at 10 and 20 μg/mL after 24, 48, and 72 h and presented on Figure 5. Cell migration takes place throughout the whole cancer growth process, but it becomes particularly significant during invasion, which is involved in the early stages of metastasis. A cancer cell is capable of migrating chemotactically and invading surrounding tissues. From the microphotographs represented on Figure 5, there is a clear depiction of dose‐dependent reduction in cancer cell migration fronts after 24, 48, and 72 h of treatment. The compound deoxysericoside (TM36) reduced cell migration at both 15 and 30 μg/mL compared to the untreated controls. After 24, 48, and 72 h of treatment, deoxysericoside (TM36) equally exhibited dose‐dependent wound healing at both 15 and 30 μg/mL when compared to the untreated control.

### 3.6 Molecular Docking Results

Molecular docking has been performed in an attempt to disclose the binding modes between terminolic acid (TM32), arjungenin (TM34), arjunglucoside I (TM35), 24‐deoxysericoside (TM36), chebuloside II (TM37), and the reference drug doxorubicin from one side and hERα and PI3Kα targets. Table 1 summarizes the free binding energies, the number of hydrogen bonds, and the number of interactions in the complexes formed between test compounds and the reference drug doxorubicin with the active residues of hERα and PI3Kα targets.

**TABLE 1:** Free binding energies, hydrogen bonding, and number of closest residues of terminolic acid (TM32), arjungenin (TM34), arjunglucoside I (TM35), 24‐deoxysericoside (TM36), chebuloside II (TM37), and the reference drug doxorubicin docked into the binding sites hERα and PI3Kα.

Compound	Free binding energy (kcal/mol)	H‐Bonds (HBs)	Number of closest residues to the docked ligand in the active site/Number of Interactions
*hERα*			
TM32	−7.40	2	THR A347, LEU A346
TM34	−5.07	1	LEU A346
TM35	−8.54	3	ASP A351, GLU A353, ARG 394
TM36	−9.04	2	GLU A353
TM37	−5.02	4	THR A347, GLU A353, ARG 394
Doxorubicin	−9.14	5	THR A347, LEU A525, ALA A350, ASP A351, LEU A536, LEU A554, TRP A383, MET A343, GLU A353, ARG 394
*PI3Kα*			
TM32	−8.37	4	GLU A849, VAL A851, TYR A836, SER A774
TM34	−6.37	5	GLU A849, VAL A851, TYR A836, SER A774, SER A919
TM35	−6.95	8	ARG A770, GLN A859, SER A919, ASP A933, TYR A836, LYS A802, SER A774
TM36	−8.84	5	GLU A849, VAL A851, SER A774, SER A919, ARG A770
TM37	−5.97	5	ARG A770, SER A854, SER A774, LYS A802, ASP A933, HIS A855
Doxorubicin	−9.58	5	SER A774, GLN A859, TRP A780, VAL A850, MET A922, VAL A851, ILE A800, GLU A849, ILE A932, ILE A848, TYR A836, ASP A933, ASP A810

**FIGURE 6:**
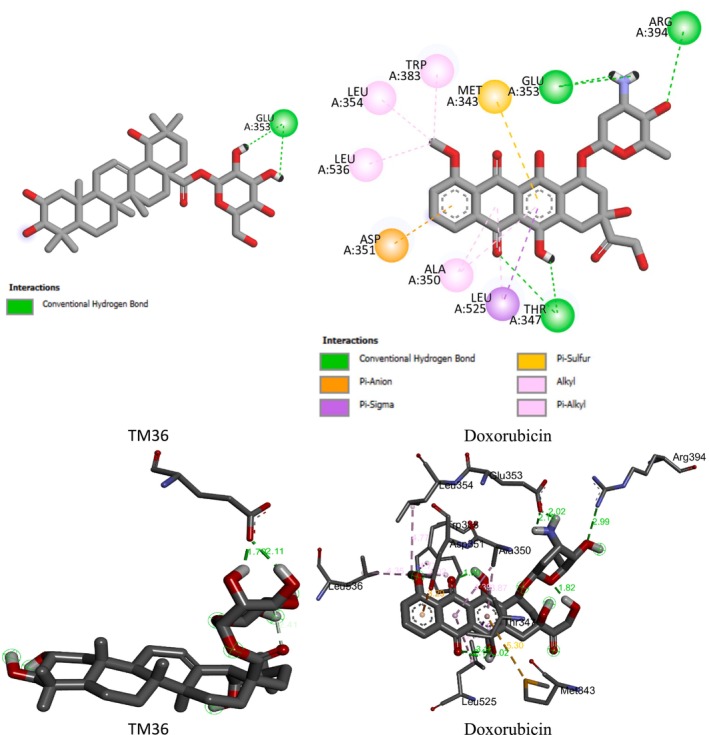
2D (up) and 3D (bottom) binding interactions of 24‐deoxysericoside (TM36) and Doxorubicin into the binding site of hERα.

**FIGURE 7:**
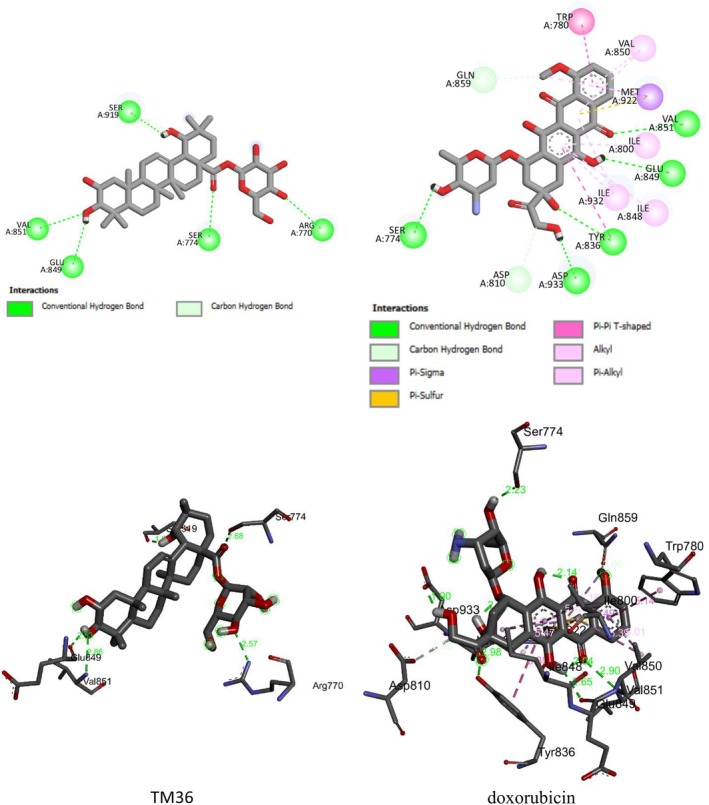
2D (up) and 3D (bottom) binding interactions of 24‐deoxysericoside (TM36) and Doxorubicin into the binding site of PI3Kα.

Molecular docking outputs reveal that terminolic acid (TM32), arjungenin (TM34), arjunglucoside I (TM35), 24‐deoxysericoside (TM36), and chebuloside II (TM37) are fitting well into the binding sites hERα and PI3Kα. The compounds from stable complexes of relative binding energies in ranges of −9.04 to −5.02 kcal mol^−1^ (hERα) and −8.84 to −5.97 kcal mol^−1^ (PI3Kα) are presented on Table 1. The negative binding energies indicate the potency of terminolic acid (TM32), arjungenin (TM34), arjunglucoside I (TM35), 24‐deoxysericoside (TM36), and chebuloside II (TM37) to inhibit hERα and PI3Kα. These inhibition processes are considered thermodynamically favorable (Table 1). For both targets, the inhibition efficiency of test samples may be strongly influenced by binding energy, the number of hydrogen bonds, and the number and types of interactions involved. This results in the formation of stable complexes between the docked compounds and amino acids of hERα and PI3Kα. The binding interactions of terminolic acid (TM32), arjungenin (TM34), arjunglucoside I (TM35), 24‐deoxysericoside (TM36), chebuloside II (TM37), and doxorubicin with hERα and PI3Kα are given in Figures S1 and S2. Figures 6 and 7 display the most stable complexes formed between the most active compound 24‐deoxysericoside (TM36) and the reference drug doxorubicin into the binding sites of hERα and PI3Kα.

### 3.7 Predicted ADME and Druglikeness

The estimated ADME and druglikeness properties of terminolic acid (TM32), arjungenin (TM34), arjunglucoside I (TM35), 24‐deoxysericoside (TM36), chebuloside II (TM37), and the reference drug doxorubicin are reported in Tables S1–S6. Except for the reference drug doxorubicin, terminolic acid (TM32), arjungenin (TM34), arjunglucoside I (TM35), 24‐deoxysericoside (TM36), and chebuloside II (TM37) obey Lipinski's rule of five. They exhibited lipophilicity values (*M*_logP_) lower than the threshold value of 4.15 (Tables S2 and S5), indicating their good druglikeness properties. TM32 and TM34 derivatives have a topological surface area (TPSA) in the range of 78–125 Å^2^, suggesting that they are likely to be orally absorbed, with bioavailability scores of 0.56 (Table S5). TM35–TM37 have TPSA out of 78–125 Å^2^, which indicates that they are unlikely to be orally absorbed (Table S1).

**FIGURE 8:**
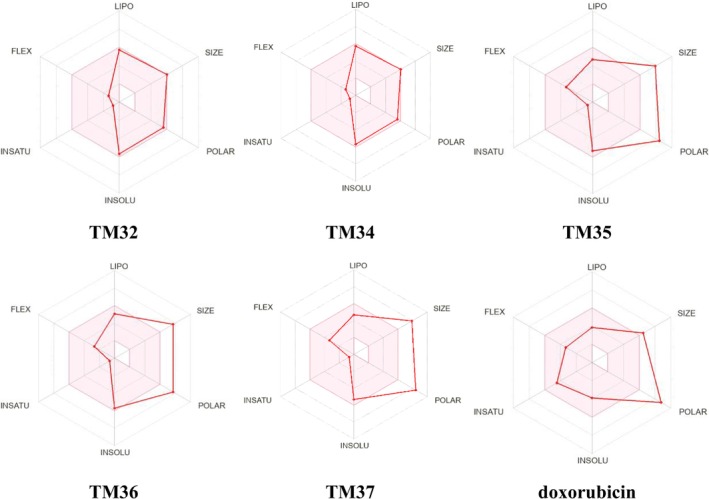
Bioavailability radars of hERα and the terminolic acid (TM32), arjungenin (TM34), arjunglucoside I (TM35), 24‐deoxysericoside (TM36), chebuloside II (TM37), and doxorubicin.

**FIGURE 9:**
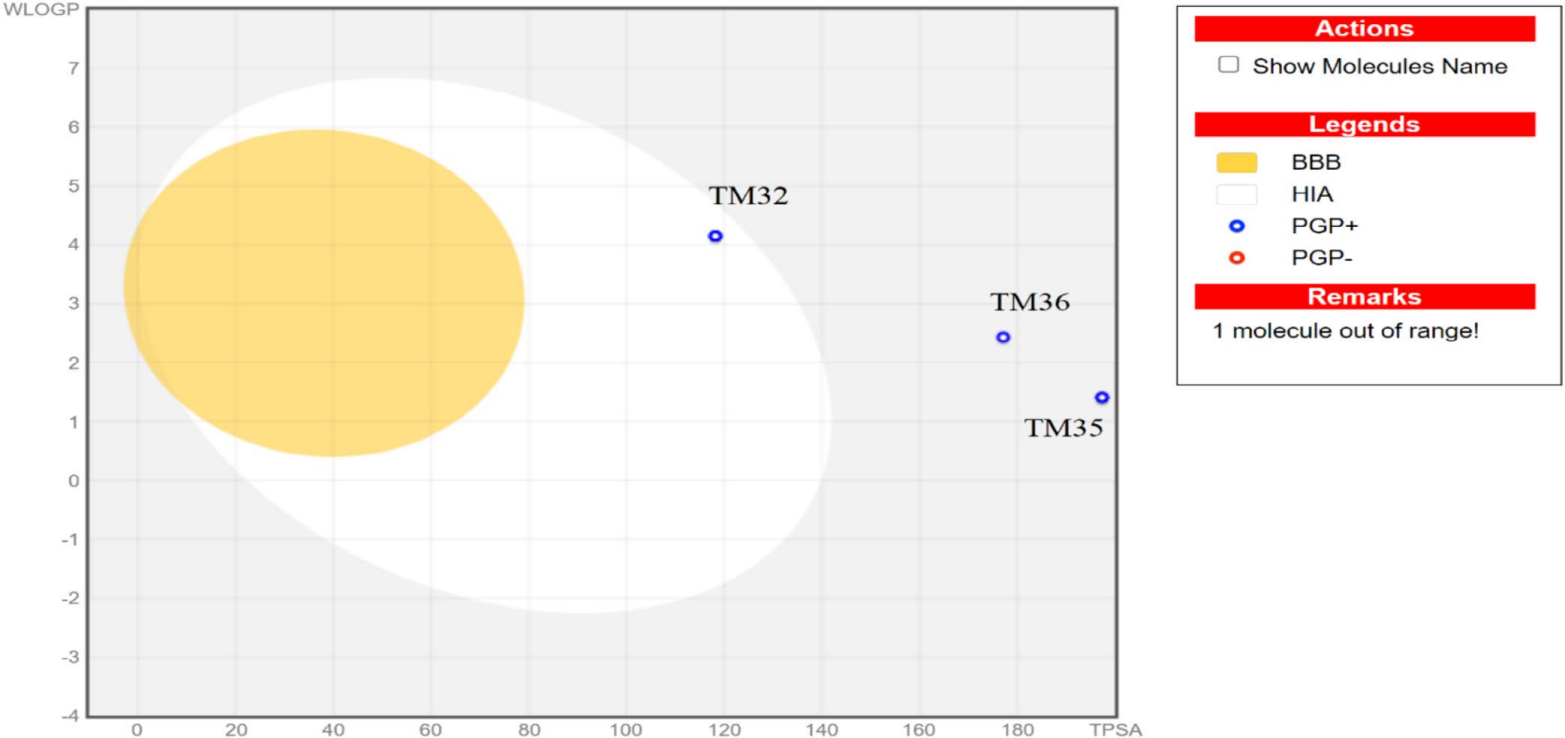
Boiled‐egg model of terminolic acid (TM32), arjungenin (TM34), arjunglucoside I (TM35), 24‐deoxysericoside (TM36), chebuloside II (TM37), and doxorubicin.

**FIGURE 10:**
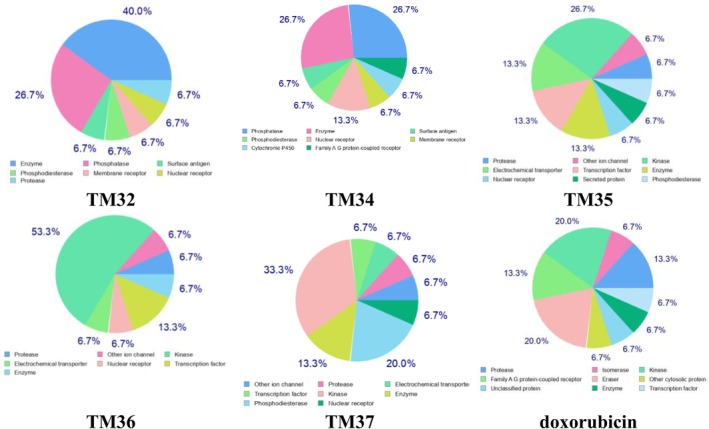
Predicted biological targets of terminolic acid (TM32), arjungenin (TM34), arjunglucoside I (TM35), 24‐deoxysericoside (TM36), chebuloside II (TM37), and doxorubicin.

The bioavailability radars of test samples are illustrated in Figure 8. Terminolic acid (TM32) and arjungenin (TM34) fall within the pink area of the polygon (Figure 8), which indicates their good oral bioavailability. The pharmacokinetic properties in Table S4 indicate that terminolic acid (TM32) and arjungenin (TM34) have high gastrointestinal (GI) absorption, while arjunglucoside I (TM35), 24‐deoxysericoside (TM36), chebuloside II (TM37), and doxorubicin have low GI absorption. The Boiled‐egg model (Figure 9) reveals that TM32 may exhibit human intestinal absorption (HIA). The probable and predicted biological targets of terminolic acid (TM32), arjungenin (TM34), arjunglucoside I (TM35), 24‐deoxysericoside (TM36), chebuloside II (TM37), and the reference drug doxorubicin are illustrated in the pie chart (Figure 10).

## 4 Discussion

Breast carcinoma is a major health problem that seriously threatens women. Treatment strategies involve multidisciplinary approaches ranging from chemotherapy to surgery and mastectomy depending on the type and stage of the cancer [29, 30]. In both metastatic and non‐metastatic breast cancers, methods involving surgery, chemotherapy, radiotherapy, gene therapy, and immunotherapy are employed [31]. Some of these methods are associated with much controversy and adverse effects. Over the past few decades, chemotherapy and medicines have played a significant role in treating advanced‐stage cancers when surgery and/or radiation therapy are not recommended for a variety of reasons [32]. Various anti‐human epidermal growth factor 2 therapeutic agents including pertuzumab, trastuzumab, trastuzumab neratinib, and emtansine are used for breast cancer treatment [33]. Chemotherapeutic agents such as platinum, anthracycline, capecitabine, taxane, or endocrine therapeutics such as tamoxifen, aromatase inhibitor, ovarian ablation/suppression, as well as bisphosphonates are recommended for breast cancer treatment [33]. There is an associated high cost for treatment of cancers with breast cancer having the highest pharmaceutical antineoplastic expenditures [34]. The activity exhibited by the compounds in this research indicates their potential as natural anticancer agents and suitable alternative chemotherapeutic substances. The terminolic acid (TM32), arjungenin (TM34), arjunglucoside I (TM35), 24‐deoxysericoside (TM36), and chebuloside II (TM37) from *Terminalia macroptera* exhibited cytotoxic effects against breast cancer cell lines. All compounds inhibited breast cancer cells, with the saponins being more potent and could be considered as starting agents for possible exploration as cytotoxic agents. Preclinical research on natural products has shown promising results to fight cancer and advance clinical research [35]. Natural compounds are also used as starting scaffolds for the development of anticancer drugs. The anticancer activity of terpenoids expressed in this study corroborates with previous investigations. Paclitaxel (Taxol), a diterpene derived from *Taxus brevifolia* , is a commercial anti‐neoplastic agent for cancer treatment which attracted interest in terpenoids as anti‐cancer therapies [36]. This suggests that the triterpenoids described in this study, with their structural similarities to some studied compounds, may be useful in the treatment of breast cancer. Triterpenoids have been shown to suppress the proliferation of MCF‐7 and MDA‐MB‐231 breast cancer cells [37]. Preclinical data have demonstrated that triterpenoids are phytochemicals with multifunctional anticancer effects, especially against breast cancers [18, 38, 39]. The anticancer properties of triterpenoids may be due to their antiproliferative effects and ability to interfere with angiogenesis and differentiation, DNA polymerase inhibition, regulation of apoptosis, impeding metastasis, and changing signal transductions [40, 41].

In this study, the most potent compound in inhibiting breast cancer is the triterpenoid saponin, deoxysericoside (TM36). Although there are no approved saponin‐based anticancer drugs, there exist reports on potent anticancer effects of saponins. The mechanism of action of saponins includes cellular invasion inhibition, antioxidant activity, ameliorating inflammation, cell‐cycle arrest, angiogenesis, induction of autophagy and apoptosis [20, 23]. Based on the screening of isolated compounds for their potential cytotoxic effect as presented on Figure 2, it is suggested that the presence of an –OH group at position 24 could reduce the cytotoxicity while glycosylation enhances it. It has been reported that saponins comprising a hydrophilic oligosaccharide moiety linked to a nonpolar triterpene skeleton exert antitumor activity through targeting multiple signaling pathways as well [21, 22]. This suggests that the saponins also interfered in signal transduction pathways that are involved in cancer treatment. The triterpenoids and saponins in this study are of the oleanane type. Antitumor effects of oleanane triterpenoids involve mechanisms such as anti‐metastasis, chemoprevention, immune stimulation, cell growth inhibition and so on [42]. In addition, the saponin deoxysericoside (TM36) inhibited cellular migration as well as colony formation. Saponins have been shown to be capable of inhibiting colony formation and cell migration of many cancer cells including breast carcinoma in a concentration‐dependent manner [43]. Observing the cytotoxic screening activity of the isolated compounds, saponins were more active than their corresponding triterpenoid aglycones. This observation is supported by the reports which depicted saponins as potent cytotoxic agents [44].

In vivo and in vitro anticancer properties can possibly be supplemented with molecular docking which gives more insights into the structure–activity relationship of the observed activity in relation to the structural features [45]. Natural products anticancer therapeutic approaches make use of synergy between in vitro, in vivo, and computational studies in the drug discovery process [46]. From the docking results, it was observed that the compounds show low binding energies though close to those of a standard anticancer drug, doxorubicin. With the human estrogen receptor alpha (hER alpha), the number of H‐bonds formed and binding energy was best for doxorubicin than isolated compounds. With the phosphatidylinositol‐3‐kinase alpha (PI3Kα) receptor, doxorubicin had the best binding energy but showed the same number of H‐bonds with the most active compound (24‐deoxysericoside). Natural compounds are seen to have favorable metabolism and pharmacokinetic properties compared to synthetic ones. The pharmacokinetic parameters such as absorption, distribution, metabolism, and excretion (ADME) properties play key roles in drug discovery and have helped to reduce the failures in clinical trials of drugs [47]. The ADME predictions and druglikeness of the compounds are reported on Tables S1–S6 using SwissADME. The pharmacokinetics, physicochemical properties, drug‐likeness, and medicinal chemistry friendliness give insights into whether the molecule will reach target in sufficient concentration and expected biological activity [48]. Some important physicochemical properties of the compounds such as molecular weights, topological polar surface area (TPSA), hydrogen bond donors, and acceptors were computed and reported on Table S1. All molecular weights were above 500 g/mol, while only terminolic acid (TM32) and arjungenin (TM34) had 5 hydrogen bond donors. Terminolic acid (TM32), arjungenin (TM34), and the most active compound 24‐deoxysericoside (TM36) had ≥ 10 hydrogen bond acceptors. As prescribed, physicochemical parameter ranges (MW ≤ 500, log *p* ≤ 5, H‐bond donors ≤ 5, H‐bond acceptors ≤ 10) are observed for over 90% of oral drugs that have passed preliminary phases of clinical trials [49]. Topological polar surface area (TPSA) reflects the totality of the polar atoms in a molecule and TPSA value ≤ 140 Å represents good oral bioavailability [50]. Only terminolic acid (TM32) and arjungenin (TM34) had TPSA values below 140 Å. The values of physicochemical parameters described are also associated with good lipophilicity and aqueous solubility of drugs. As concerns lipophilicity, log *p* ≤ 5 is required, implying that all compounds demonstrated log P values within this range as shown on Table S2. Drug molecules are expected to be soluble so that they can be absorbed into the bloodstream and also permeate biological membranes. For permeation, drugs must have lipophilic groups and an optimal partition coefficient (LogP), thereby requiring a balance between lipophilicity and hydrophilicity [51]. Estimated solubility (ESOL Log S) is a model that predicts the water solubility of a compound based on its molecular structure and when it falls in the range −4 to −2 it means that the compound is soluble and moderately soluble when between −6 and −4 [52]. This means that the compounds were overall, soluble as indicated in Table S3. Table S4 indicates that the compounds have high to low gastrointestinal (GI) absorption and none of them can cross the blood‐brain barrier (BBB). It also predicted that the compounds can use the P‐glycoprotein (P‐gp) transporter as a means of absorption and excretion. No prediction of the inhibition of cytochrome or impairment of enzymes that metabolize drugs in the intestines and liver. The skin permeability (*K*_p_) measured as log *K*_p_ (cm/s), represents the rate of penetration of membranes by molecules and a negative log *K*_p_ value indicates reduced permeation [53]. All compounds showed negative values of log *K*_p_ indicating their poor permeation. The computed druglikeness of the compounds are presented on Table S5. The compounds arjunglucoside I (TM35), 24‐deoxysericoside (TM36), and chebuloside II (TM37) and doxorubicin showed at least a violation of all five filters (Lipinski, Ghose, Veber, Egan, and Muegge) used in the prediction. The other compounds terminolic acid (TM32) and arjungenin (TM34) showed violations of Lipinski (one violation) and Ghose (three violations) filters. Bioavailability refers to the extent a substance or drug becomes completely available to its intended biological destination or the fraction of the active form of a drug that reaches systemic circulation unaltered [54, 55]. The score of bioavailability is usually expressed in a decimal range (between 0 and 1). The higher the value, the greater the oral bioavailability that the compound has [56]. According to Table S5, terminolic acid (TM32) and arjungenin (TM34) had higher bioavailability of 0.56 compared to arjunglucoside I (TM35), 24‐deoxysericoside (TM36), chebuloside II (TM37), and doxorubicin which all had bioavailability values of 0.17. The BRENK and PAIN alerts screening of the compounds are provided on Table S6. No compound showed a PAIN alert except doxorubicin which showed a single alert. This indicates that the isolated compounds do not have dangerous structural features and could possibly exert physiological activity as revealed from the molecular docking [57, 58]. However, all compounds showed one BRENK alert each. The presence of BRENK alerts suggests that some of the structural fragments can be unstable, toxic, or chemically reactive [59]. In all, molecular docking gave structure–activity insights while ADME evaluations predicted how the compounds may be absorbed, distributed, metabolized, and excreted by body organs [60].

## 5 Conclusions

*Terminalia macroptera* has proven its medicinal efficacy in various traditional medicine preparations; however, there is a need for scientific studies to validate its use in ethnomedicines. In this study, triterpenoids including terminolic acid (TM32), arjungenin (TM34), arjunglucoside I (TM35), 24‐deoxysericoside (TM36), and chebuloside II (TM37) from *T. macroptera* were evaluated for their anticancer properties. The compounds displayed cytotoxicity against estrogen non‐sensitive (MDA‐MB 231) and estrogen sensitive (MCF‐7) breast cancer cell lines, with deoxysericoside (TM36) being the most active. This compound showed antiproliferative effects, being able to reduce cellular migration of cancer cells. This implies that the compound could stop metastasis of the breast carcinoma. The study was supplemented with molecular docking and ADME predictions, which showed their binding effectiveness into receptor site of both hERα and PI3Kα with appropriate druglikeness. The studies showed that deoxysericoside (TM36) could be used for development of anticancer therapies, requiring further experiments.

## Author Contributions

**Romeo Toko Feunaing:** methodology, investigation, formal analysis, writing – original draft, validation. **Cyrille Leonel Tchuente Djoko:** methodology, investigation, validation, formal analysis. **Alfred Ngenge Tamfu:** conceptualization, methodology, software, investigation, validation, formal analysis, writing – review and editing, writing – original draft, data curation. **Stephane Zingue:** conceptualization, methodology, validation, investigation, data curation, formal analysis, writing – original draft, writing – review and editing. **Abel Joel Yaya Gbaweng:** methodology, investigation, formal analysis, validation. **Emmanuel Talla:** conceptualization, data curation, investigation, methodology, validation, formal analysis. **El Hassane Anouar:** conceptualization, methodology, investigation, software, formal analysis, writing – original draft, writing – review and editing.

## Funding

This work was partially funded by the Organization for the Prohibition of Chemical Weapons (Grant No. OPCW 188/22 1/3 INS).

## Ethics Statement

The authors have nothing to report.

## Consent

The authors have nothing to report.

## Conflicts of Interest

The authors declare no conflicts of interest.

## Supporting information


**Data S1:** Supporting Information.
**Figure S1:** 3D and 2D binding interactions of terminolic acid (TM32), arjungenin (TM34), arjunglucoside I (TM35), 24‐deoxysericoside (TM36), chebuloside II (TM37) and the reference drug doxorubicin into the binding site of human estrogen receptor‐α (hERα).
**Figure S2:** 3D and 2D binding interactions of terminolic acid (TM32), arjungenin (TM34), arjunglucoside I (TM35), 24‐deoxysericoside (TM36), chebuloside II (TM37) and the reference drug doxorubicin into the binding site of phosphatidylinositol 3‐kinase (PI3K).
**Figure S3:** ^1^H and ^13^C NMR spectra of Arjungenin (TM34).
**Figure S4:** ^1^H and ^13^C NMR spectra of Terminolic acid (TM32).
**Figure S5:** ^1^H and ^13^C NMR spectra of 24‐Deoxysericoside (TM36).
**Figure S6:** ^1^H and ^13^C NMR spectra of Arjunglucoside I (TM35).
**Figure S7:** ^1^H and ^13^C NMR spectra of Chebuloside II (TM37).
**Table S1:** Physicochemical Properties of 24‐deoxysericoside (TM32, TM34‐TM37) and the reference drug doxorubicin.
**Table S2:** Lipophilicity of 24‐deoxysericoside (TM32, TM34‐TM37) and the reference drug doxorubicin.
**Table S3:** Water Solubility of 24‐deoxysericoside (TM32, TM34‐TM37) and the reference drug doxorubicin.
**Table S4:** Pharmacokinetics properties of 24‐deoxysericoside (TM32, TM34‐TM37) and the reference drug doxorubicin.
**Table S5:** Druglikeness Properties of 24‐deoxysericoside (TM32, TM34‐TM37) and the reference drug doxorubicin.
**Table S6:** Medicinal Properties of 24‐deoxysericoside (TM32, TM34‐TM37) and the reference drug doxorubicin.

## Data Availability

The data that support the findings of this study are available from the corresponding author upon reasonable request.
