# Supplementary material for: Antiproliferative Effect of 24‐Deoxysericoside From Terminalia macroptera Guill. & Perr. (Combretaceae) Against Breast Carcinoma: In Vitro, Molecular Docking and ADME Assessment
Source: Cancer Rep (Hoboken). 2026 Apr 7;9(4):e70541. doi: 10.1002/cnr2.70541 (PMC13056511; doi:10.1002/cnr2.70541)
Supplement: Supplementary file 1 — Data S1: Supporting Information. Figure S1: 3D and 2D binding interactions of terminolic acid (TM32), arjungenin (TM34), arjunglucoside I (TM35), 24‐deoxysericoside (TM36), chebuloside II (TM37) and the reference drug doxorubicin into the binding site of human estrogen receptor‐α (hERα). Figure S2: 3D and 2D binding interactions of terminolic acid (TM32), arjungenin (TM34), arjunglucoside I (TM35), 24‐deoxysericoside (TM36), chebuloside II (TM37) and the reference drug doxorubicin into the binding site of phosphatidylinositol 3‐kinase (PI3K). Figure S3: 1H and 13C NMR spectra of Arjungenin (TM34). Figure S4: 1H and 13C NMR spectra of Terminolic acid (TM32). Figure S5: 1H and 13C NMR spectra of 24‐Deoxysericoside (TM36). Figure S6: 1H and 13C NMR spectra of Arjunglucoside I (TM35). Figure S7: 1H and 13C NMR spectra of Chebuloside II (TM37). Table S1: Physicochemical Properties of 24‐deoxysericoside (TM32, TM34‐TM37) and the reference drug doxorubicin. Table S2: Lipophilicity of 24‐deoxysericoside (TM32, TM34‐TM37) and the reference drug doxorubicin. Table S3: Water Solubility of 24‐deoxysericoside (TM32, TM34‐TM37) and the reference drug doxorubicin. Table S4: Pharmacokinetics properties of 24‐deoxysericoside (TM32, TM34‐TM37) and the reference drug doxorubicin. Table S5: Druglikeness Properties of 24‐deoxysericoside (TM32, TM34‐TM37) and the reference drug doxorubicin. Table S6: Medicinal Properties of 24‐deoxysericoside (TM32, TM34‐TM37) and the reference drug doxorubicin. [file CNR2-9-e70541-s001.docx]

**Antiproliferative effect of 24-Deoxysericoside from *Terminalia macroptera* Guill. & Perr. (Combretaceae) against breast carcinoma: *in vitro,* molecular docking and ADME assessment**

Romeo Toko Feunaing^1^, Alfred Ngenge Tamfu^2^*, Abel Joel Yaya Gbaweng^1^, Cyrille Leonel Tchuente Djoko^1^, Emmanuel Talla^1,3^, El Hassane Anouar^4^, Stephane Zingue^5^*

*^1^Department of Chemistry, Faculty of Sciences, University of Ngaoundere, P.O. Box 454, Ngaoundere, Cameroon.*

*^2^Department of Chemical Engineering, School of Chemical Engineering and Mineral Industries, University of Ngaoundere, P.O. Box 454 Ngaoundere, Cameroon.*

*^3^Department of Materials Engineering, School of Chemical Engineering and Mineral Industries, University of Ngaoundere, P.O. Box 454 Ngaoundere, Cameroon.*

*^4^Department of Chemistry, College of Sciences and Humanities in Al-Kharj, Prince Sattam bin Ab-dulaziz University, Al-Kharj, Saudi Arabia.*

*^5^Department of Pharmacotoxicology and Pharmacokinetics, Faculty of Medicine and Biomedical Sciences, University of Yaounde 1, P.O. Box 1364, Yaounde, Cameroon.*

*** Corresponding authors**

Alfred Ngenge Tamfu: [macntamfu@yahoo.co.uk](mailto:macntamfu@yahoo.co.uk)

Stephane Zingue: [stephane.zingue@fmsb-uy1.cm](mailto:stephane.zingue@fmsb-uy1.cm)

**Abstract**

**Background and objectives:** *Terminalia macroptera* (Combretaceae) is an important medicinal plant in the traditional pharmacopeoa in most tropical areas, where its different parts are used in treating illnesses. In this study, two oleanane-type triterpenoids; terminolic acid (TM32) and arjungenin (TM34), together with three saponins; arjunglucoside I (TM35), 24-deoxysericoside (TM36) and chebuloside II (TM37) from *T. macroptera*, were screened for their cytotoxic effects against breast cancer cell lines.

**Methods:** The compounds were isolated using column chromatography and characterized from their NMR data. Their cytotoxic and antiproliferative effects against estrogen non-sensitive (MDA-MB 231) and estrogen sensitive (MCF-7) breast cancer cell lines were evaluated.

**Results:** Against estrogen non-sensitive (MDA-MB 231) cancer cell lines, deoxysericoside (TM36) was profoundly active compared to the control. Terminolic acid (TM32), arjungenin (TM34) and arjunglucoside I (TM35) were also active. Against estrogen sensitive (MCF-7) breast cancer cell lines, deoxysericoside (TM36) exhibited significant activity (*p* < 0.05) compared to control experiments. The most active compound had an optimum concentration of 30 µg/mL. Deoxysericoside (TM36) showed concentration-dependent inhibition percentages at 15 and 30 µg/mL and MDA-MB 231 breast carcinoma cells were more susceptible. MDA-MB 231 treated with 15 and 30 µg/mL of deoxysericoside (TM36), showed significant reduction (*p* < 0.05) in clone formation after 48 hours when compared to untreated controls, suggesting that it can restrict cancer to a preliminary stage. The deoxysericoside (TM36) reduced cell migration with dose-dependent improvement in wound healing at 15 and 30 µg/mL, revealed by the micrographs. Molecular docking indicated that the compounds fit well into hERα and PI3Kα receptor binding sites, forming stable complexes with binding energies in ranges of -9.04 to -5.02 kcal mol^-1^ (hERα receptor) and –8.84 to -5.97 kcal mol^-1^ (PI3Kα receptor). The compounds exhibited appreciable drug likeness predicted using SwissADME.

**Conclusion:** The studies showed that the isolated compounds could be used for the development of anticancer therapies.

**Keywords:** *Terminalia macroptera,* triterpenoid saponins, deoxysericoside, breast cancer, molecular docking

**Contents**

- Figure S1: 3D and 2D binding interactions of terminolic acid (TM32), arjungenin (TM34), arjunglucoside I (TM35), 24-deoxysericoside (TM36), chebuloside II (TM37) and the reference drug doxorubicin into the binding site of human estrogen receptor-α (hERα).
- Figure S2: 3D and 2D binding interactions of terminolic acid (TM32), arjungenin (TM34), arjunglucoside I (TM35), 24-deoxysericoside (TM36), chebuloside II (TM37) and the reference drug doxorubicin into the binding site of phosphatidylinositol 3-kinase (PI3K).
- Figure S3 : ^1^H and ^13^C NMR spectra of Arjungenin (TM34)
- Figure S4 : ^1^H and ^13^C NMR spectra of Terminolic acid (TM32)
- Figure S5 : ^1^H and ^13^C NMR spectra of 24-Deoxysericoside (TM36)
- Figure S6 : ^1^H and ^13^C NMR spectra of Arjunglucoside I (TM35)
- Figure S7 : ^1^H and ^13^C NMR spectra of Chebuloside II (TM37)
- NMR data of isolated compounds
- Table S1: Physicochemical Properties of 24-deoxysericoside (TM32, TM34-TM37) and the reference drug doxorubicin.
- Table S2: Lipophilicity of 24-deoxysericoside (TM32, TM34-TM37) and the reference drug doxorubicin.
- Table S3: Water Solubility of 24-deoxysericoside (TM32, TM34-TM37) and the reference drug doxorubicin.
- Table S4: Pharmacokinetics properties of 24-deoxysericoside (TM32, TM34-TM37) and the reference drug doxorubicin.
- Table S5: Druglikeness Properties of 24-deoxysericoside (TM32, TM34-TM37) and the reference drug doxorubicin.
- Table S6: Medicinal Properties of 24-deoxysericoside (TM32, TM34-TM37) and the reference drug doxorubicin.

# Figure S1: 3D and 2D binding interactions of terminolic acid (TM32), arjungenin (TM34), arjunglucoside I (TM35), 24-deoxysericoside (TM36), chebuloside II (TM37) and the reference drug doxorubicin into the binding site of human estrogen receptor-α (hERα).

| TM32 | 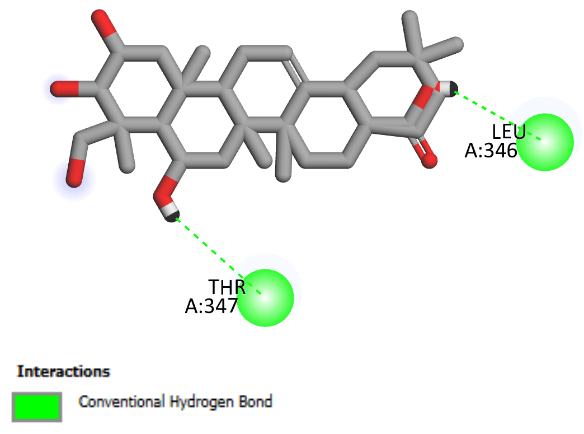 | 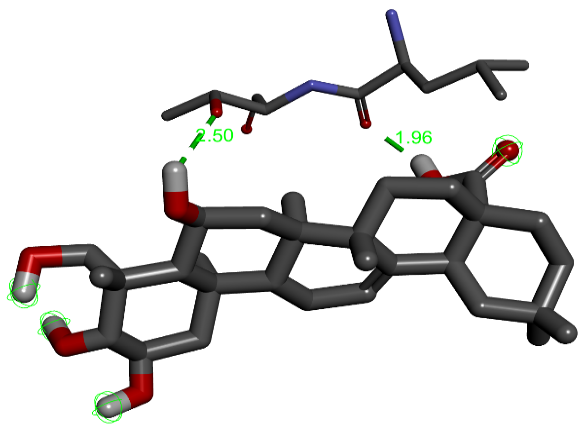 |
| --- | --- | --- |
| TM34 | 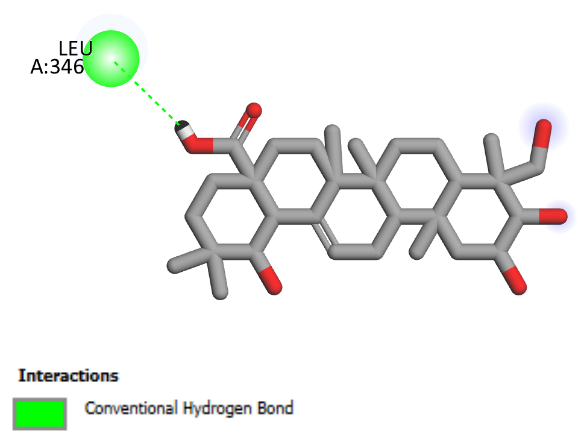 | 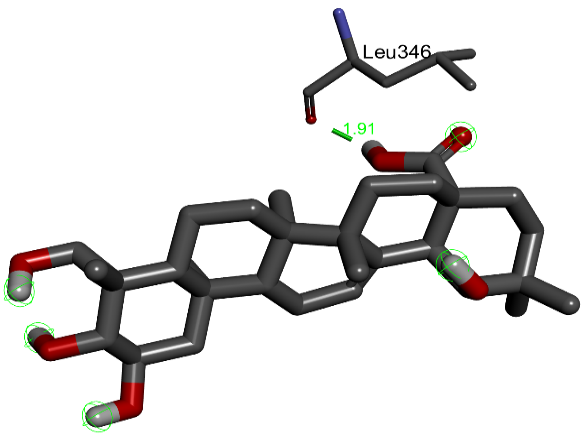 |
| TM35 | 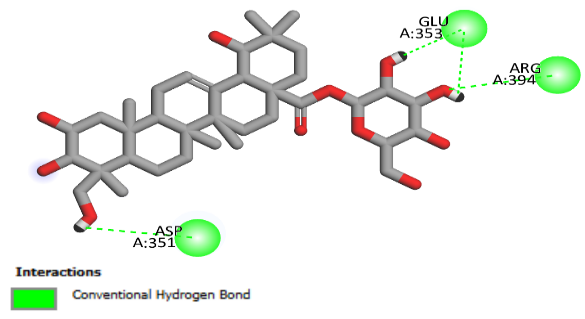 | 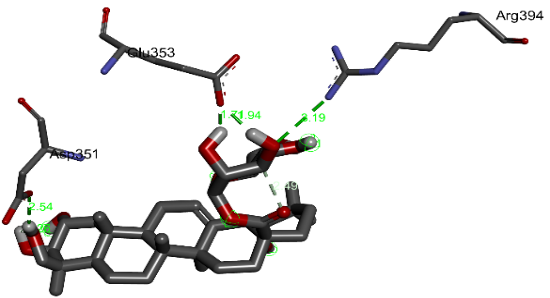 |
| TM36 | 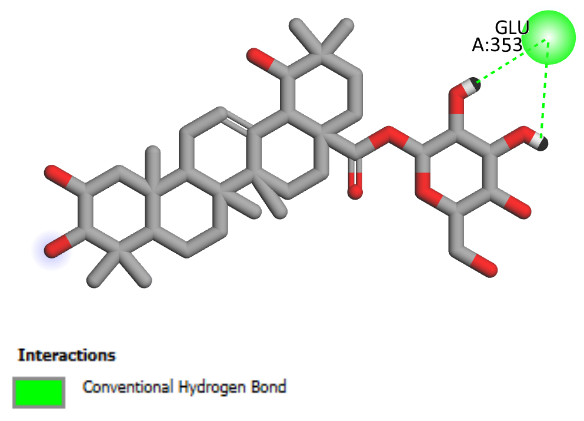 | 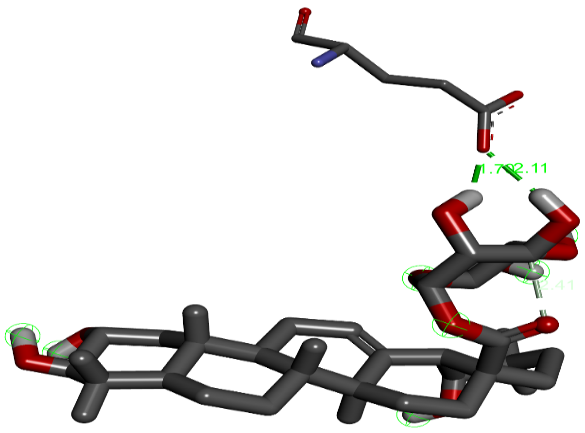 |
| TM37 | 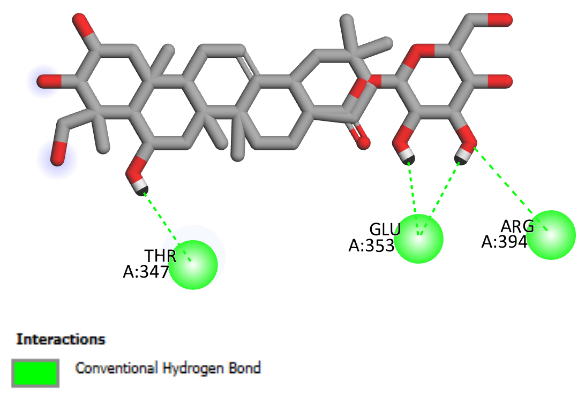 | 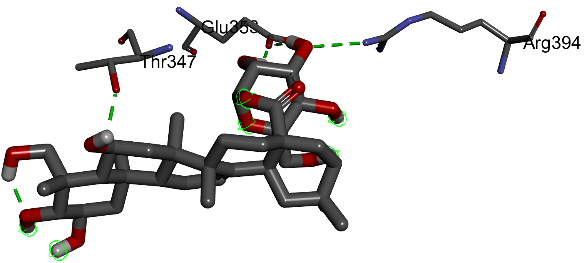 |
|  |  |  |
| Doxorubicin | 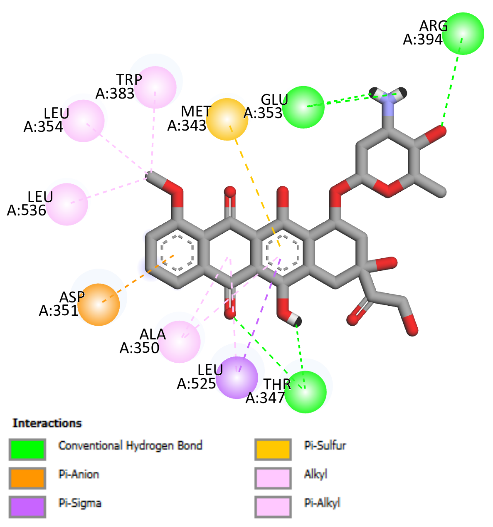 | 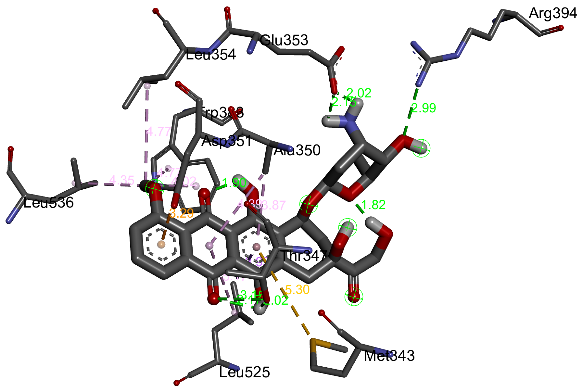 |

# Figure S2: 3D and 2D binding interactions of terminolic acid (TM32), arjungenin (TM34), arjunglucoside I (TM35), 24-deoxysericoside (TM36), chebuloside II (TM37) and the reference drug doxorubicin into the binding site of phosphatidylinositol 3-kinase (PI3K).

| TM32 | 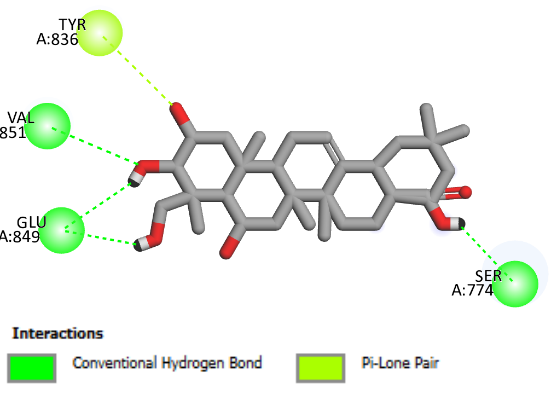 | 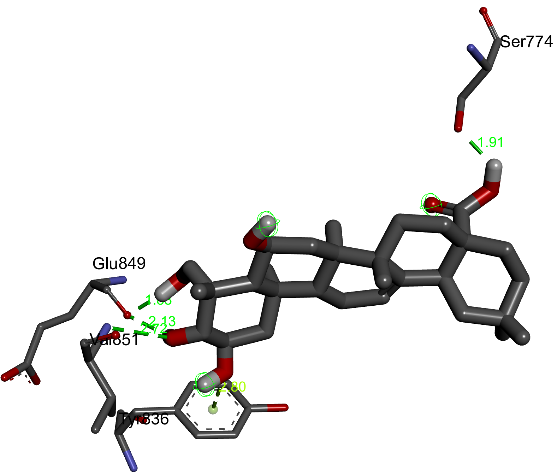 |
| --- | --- | --- |
| TM34 | 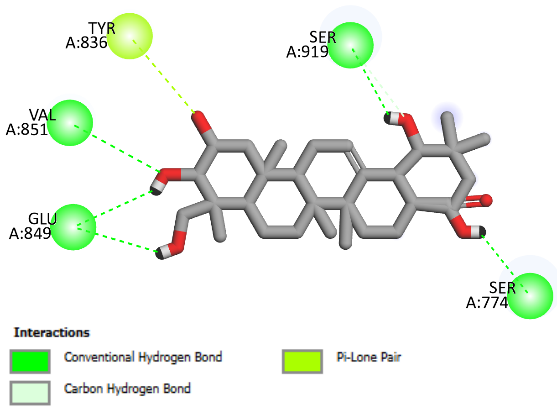 | 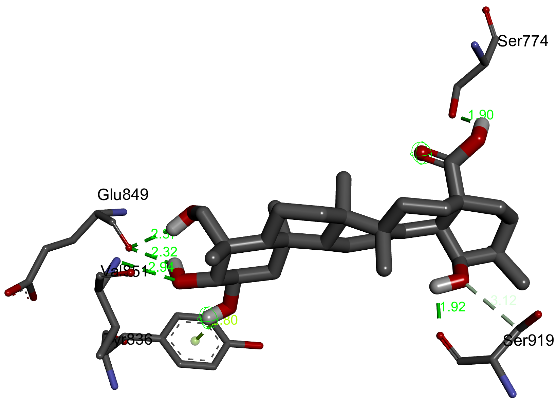 |
| TM35 | 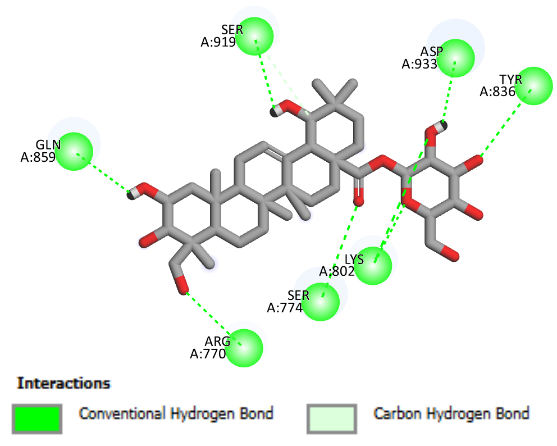 | 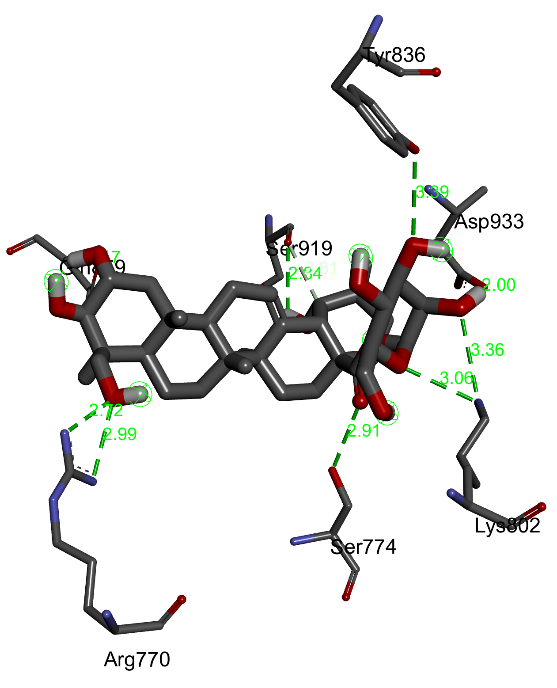 |
| TM36 | 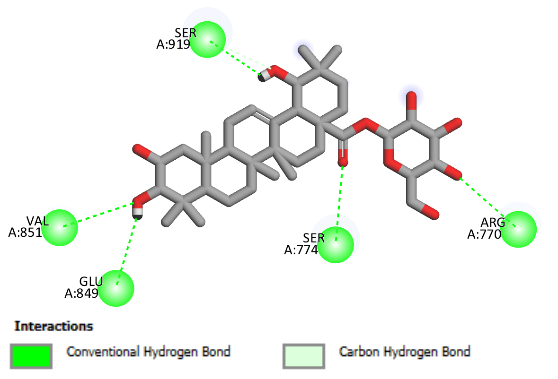 | 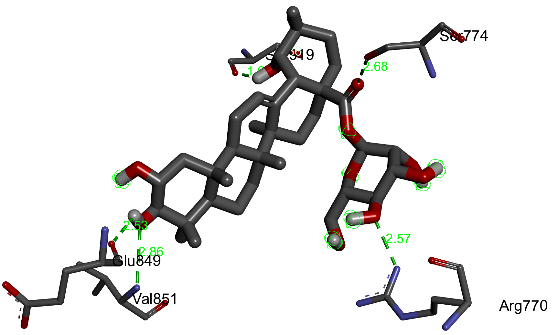 |
| TM37 | 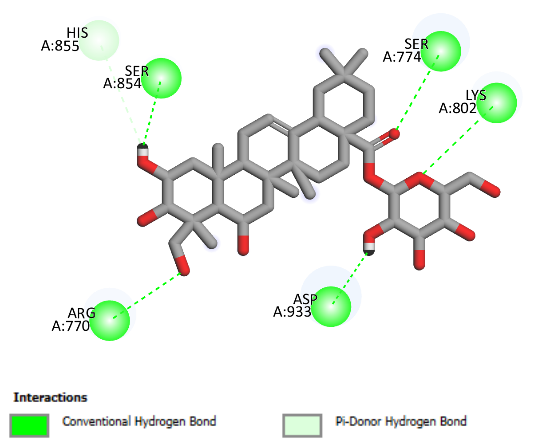 | 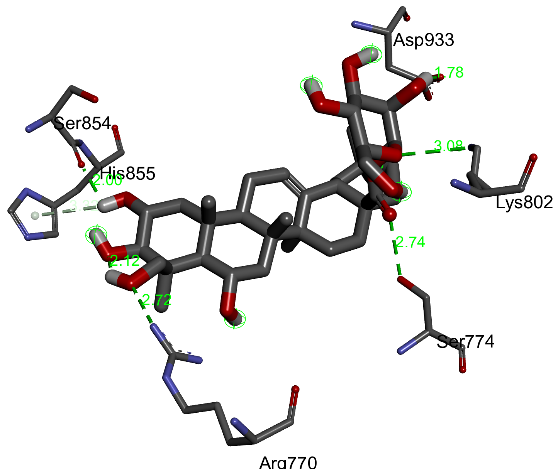 |
| doxorubicin | 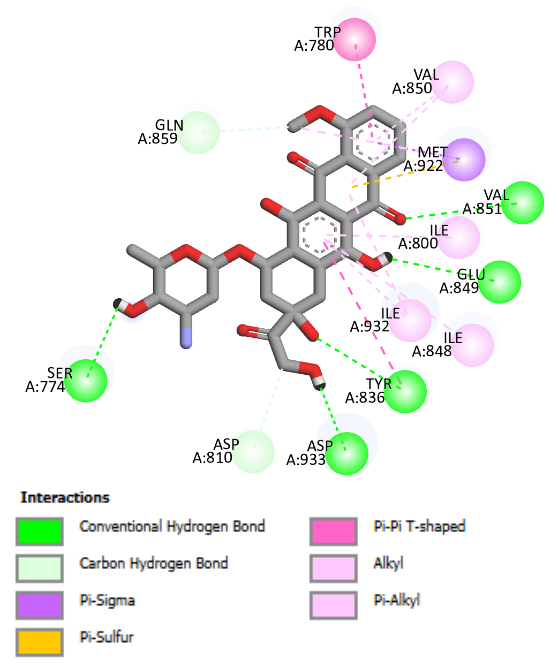 | 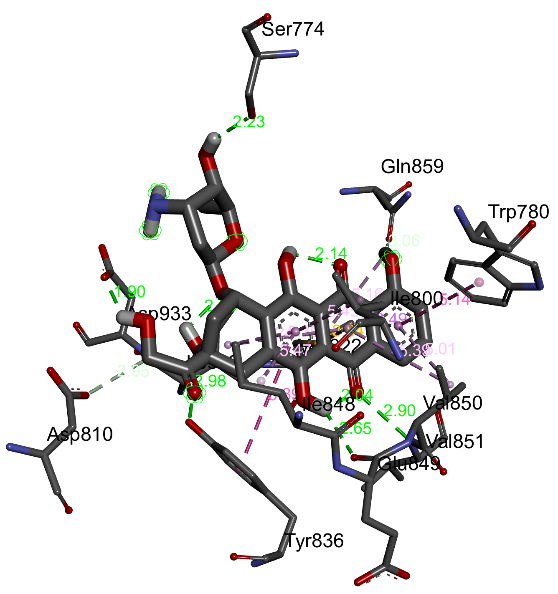 |

| **Figure S3 : ^1^H and ^13^C NMR spectra of Arjungenin (TM34)** |
| --- |
|   **^1^H NMR TM34**   |
|   **^13^C NMR TM34**   |
| **Figure S4 : ^1^H and ^13^C NMR spectra of Terminolic acid (TM32)** |
|   **^1^H NMR TM32**   |
|   **^13^C NMR TM32**    **Figure S5 : ^1^H and ^13^C NMR spectra of 24-Deoxysericoside (TM36)** |
|   **1H NMR TM36**   |
|   **^13^C NMR TM36**   |
| **Figure S6 : ^1^H and ^13^C NMR spectra of Arjunglucoside I (TM35)** |
|   **^1^H NMR TM35**   |
|   **^13^C NMR TM35**   |
| **Figure S7 : ^1^H and ^13^C NMR spectra of Chebuloside II (TM37)** |
|   **^1^H NMR TM37**   |
|   **^13^C NMR TM37**   |

***NMR data of isolated compounds***

**Arjungenin (TM34)**: **^1^H NMR (CD_3_OD, 500 MHz)**: ***δ***H ppm 5.37 (1H, t, *J****=*** 3.3 Hz, H-12), 3.71 (1H, *td*, *J****=*** 11.3, 4.4 Hz, H-2), 3.35 (1H, *d*, *J****=*** 9.6 Hz, H-3), 3.36 (1H, d, *J****=*** 11.2 Hz, H-24a), 3.27 (1H, d, *J****=*** 11.7 Hz, H-24b), 3.28 (1H, *d*, *J****=*** 3.8 Hz, H-19), 3.07 (1H, *br*, H-18), 1.33 (1H, *m*, H-5), 1.29 (3H, *s*, H-27), 1.01 (3H, *s*, H-25), 0.94 (3H, *s*, H-29), 0.97 (3H, *s*, H-30), 0.76 (3H, *s*, H-26), 0.72 (3H, *s*, H-23), 2.5-1.0 (alicyclic protons). **^13^C NMR (CD_3_OD, 125** **MHz):** *δ***_C_** 181.3 **(C-28),** 143.1 **(C-13),** 123.7 **(C-12),** 81.2 **(C-19),** 77.8 **(C-3),** 68.3 **(C-2),** 66.3 **(C-23),** 47.7 **(C-9),** 47.5 **(C-5),** 46.1 (C-1), 45.2 (C-17), 43.7 (C-18), 42.6 (C-4), 41.3 (C-14), 39.4 (C-8), 37.9 (C-10), 34.6 (C-20), 32.6 (C-7), 28.1 (C-21), 31.9 (C-6), 28.0 (C-15), 27.3 (C-16), 25.5 (C-30), 24.1 (C-29), 24.1 (C-27), 23.6 (C-11), 18.0 (C-22), 16.6 (C-26), 16.4 (C-25), 12.6 (C-23).

**Terminolic acid (TM32)**. **^1^H NMR (CD_3_OD, 500 MHz)**: 5.30 (lH, *t, J* ***=*** 3.46 *Hz,* H-12), 4.41 (lH, ***bs*,** H-6), 3.75 (lH, **m**, H-2), 3.60 (lH, d, ***J* *=*** 11.1 *Hz,* H-23), 3.46 (lH, d, ***J* *=*** 11.1 *Hz,* H-23), 3.31 (lH, *d, J* ***=*** 9.7 *Hz,* H-3), 2.89 (lH, *dd,* ***J =*** 13.5, 3.7 *Hz,* H-18), 1.40 (3H, *s***,** CH_3_-25), 1.08 (3H, *s***,** CH_3_-24), 1.11 (3H, *s* ***,*** CH_3_-26), 1.16 (3H, *s***,** CH_3_-27), 0.93 (3H, *s***,** CH_3_-29), 0.96 (3H, **s,** CH_3_-30), 2.5-1.0 (alicyclic protons). **^13^C-NMR** (125 MHz, CD_3_OD in ppm): 180.6 (C-28), 143.2 (C-13), 122.3 (C-12), 76.8 (C-3), 68.2 (C-2), 67.1 (C-6), 64.5 (C-23), 48.1 (C-1), 48.1 (C-5), 47.6 (C-9), 46.2 (C-17), 48.8 (C-19),43.3 (C-4), 42.1 (C-14), 41.3 (C-18), 39.7 (C-7), 38.3 (C-8), 37.1 (C-l0), 33.5 (C-21), 32.4 (C-29), 32.2 (C-22), 30.2 (C-20), 27.4 (C-15), 25.1 (C-27), 23.2 (C-11), 22.7 (C-30), 22.6 (C-16), 17.6 (C-25), 17.4 (C-26), 13.8 (C-24).

**24-Deoxysericoside (TM36).** **^1^H NMR** (CD_3_OD, 500 MHz): ***δ***_H_ ppm: 5.39 (lH, *d* ***,*** *J* *=* 8.2 Hz*,* H-l'), 5.35 (lH, t, *j* *=* 3.3 Hz, H-12), 4.44 (lH, m, H_a_-6'), 4.39 (lH, *m*, H_b_-6'), 3.64 (lH, *t, J=* 8.90 *Hz,*H-4'), 3.37 (lH, t , *J =* 8.85 Hz, H-3'), 3.35 (lH, *t, J =* 8.50 *Hz,* H-2'), 3.32 (lH, *m*, H-2), 3.31 (lH *m*, H-5'), 3.28 (lH, *d, J* *=* 3.29*Hz,*H-19), 3.07 (lH, ***bs,*** H-18), 2.91 (lH, *d, J* *=* 9.46 *Hz,* H-3), 1.31 (3H,s, CH_3_-27), 1.03 (3H,s, CH_3_-23), 1.01(3H, **S,** CH_3_,-26), 0.96 (3H, *s* ***,*** CH_3_- 29), 0.95 (3H,**s,**CH_3_-24), 0.82 (3H, **s,** CH_3_-25), 0.76 (3H,**s,**CH_3_-30), 2.8-1.0 (alicyclic protons). **^13^C -NMR** (CD_3_OD, 500 MHz**)**: 177.3 (C-28), 144.1 (C-13), 123.4 (C-12), 94.5 (C-l'), 83.3 (C-3), 81.0 (C-19),77.3 (C-5'), 76.59 (C-3'), 72.5 (C-2'), 69.6 (C-4'), 68.1 (C-2),60.9 (C-6'), 54.4 (C-5), 48.4 (C-9), 47.8 (C-l), 46.6 (C-17), 45.7 (C-18), 43.6 (C-14), 41.2 (C-8), 39.5 (C-4), 39.1 (C-l0), 34.5 (C-20), 32.4 (C-7), 31.8 (C-22), 28.1 (C-23), 28.0 (C-15), 27.8 (C-21), 27.2 (C-29), 27.0 (C-16), 23.7 (C-27), 23.5 (C-30), 23.5 (C-11), 18.3 (C-6), 16.3 (C-26), 15.9 (C-24), 15.6 (C-25).

**Arjunglucoside I (TM35)**. **^1^H NMR (CD_3_OD, 500 MHz** in ppm**)**: 5.41 (lH, *d, J* ***=*** 8.2 Hz, H-1'), 5.33 (lH, *t, J****=*** 3.31 Hz, H-12), 3.82 (lH, *dd, J* ***=*** 12.03, 1.4 Hz, H-6'), 3.71 (lH, ***td,* 12.5, 4.2** Hz, H-2), 3.70 (lH, dd, ***J =*** 12.1, 4.1 Hz, H-6'), 3.39 (lH, d, ***J =*** 11.1 Hz, H-23), 3.36 (lH, d, ***J =*** 11.1 Hz, H-23), 3.41 (lH, *t, J* ***=*** 8.7 Hz, H-3'), 3.37 (lH, *t, J* ***=*** 8.4 Hz, H-5'), 3.37 (lH, , *t ,*J ***=*** 8.48 Hz**,** H-4'), 3.36 (lH, *d, J* ***=*** 1.5 Hz, H-3), 3.33 (lH, *t ,*J ***=*** 8.8 Hz, H-2'), 3.07 (lH, brs, H-18), 1.32 (3H, *s,* CH_3_-27), 1.04 (3H, ***S,*** CH_3_-25), 0.76 (3H, *s ,* CH_3_-26), 0.72 (3H, *s****,*** CH_3_-24), 0.93 (3H, *s****,*** CH_3_-29), 0.96 (3H, *s,* CH_3_-30), 2.5-1.0 (alicyclic protons). **^13^C-NMR** (**CD_3_OD, 500 MHz** in ppm): 177.8 (C-28), 144.3 (C-13), 123.7 (C-12), 94.8 (C-1'), 81.9 (C-19), 78.7 (C-5'), 77.9 (C-3'), 78.7 (C-3), 73.5 (C-2'), 70.5 (C-4'), 69.6 (C-2), 62.3 (C-6'), 66.6 (C-23), 47.9 (C-9), 47.5 (C-1), 47.6 (C-5), 46.7 (C-17), 44.1 (C-4), 41.3 (C-14), 40.9 (C-18), 40.0 (C-8), 39.2 (C-7), 38.5 (C-l0), 31.2 (C-21), 32.0 (C-29), 29.2 (C-22), 36.0 (C-20), 29.3 (C-15), 28.7 (C-27), 25.1 (C-11), 24.8 (C-30), 26.6 (C-16), 19.7 (C-6), 17.8 (C-25), 17.1 (C-26), 13.8 (C-24).

**Chebuloside II (TM37). ^1^H NMR (CD_3_OD, 500 MHz)**: 5.39 (lH, d, J ***=*** 8.1 Hz, H-1'), 5.32 (lH, *t*, *J* ***=*** 3.32 Hz, H-12), 4.38 (lH, ***bs,*** H-6), 4.43 (lH, dd, *J* ***=*** 11.67, 2.5Hz, H-23), 3.74 (lH, ***m***, H-2), 3.67 (lH, dd, ***J =*** 11.67, 4.01Hz, H-23), 3.61 (lH, d, J ***=*** 10.6 Hz, H-23), 3.43 (lH, *m*, H-3'), 3.42 (lH, ***bs***, H-23), 3.36 (lH, **m,** H-5'), 3.36 (lH, **m,** H-4'), 3.33(lH, *t,* J ***=*** 8.51 Hz, H-2'), 3.30 (lH, *bs*, ,H-3), 2.89 (lH, dd, ***J =*** 12.40, 3.77 Hz, H-18), 1.40 (3H, *s,* CH_3_-25), 1.16 (3H, ***s,*** CH_3_-24), 1.09 (3H, *s ,* CH_3_-26), 1.08 (3H, ***s,*** CH_3_-27), 0.96 (3H, ***s,*** CH_3_-29), 0.93 (3H, *s,* CH_3_-30), 25-1.0 (alicyclic protons). **^13^C NMR (CD_3_OD, 125 MHz)**: 176.6 (C-28), 142.9 (C-13), 122.4 (C-12), 94.4 (C-1'), 77.2 (C-5'), 76.9 (C-3'), 76.7 (C-3), 72.5 (C-2'), 69.7 (C-4'), 68.2 (C-2), 67.1 (C-6), 64.4 (C-6'), 61.0 (C-23), 48.7 (C-1),48.0 (C-5), 47.5 (C-9), 46.6 (C-17), 46.3 (C-19), 45.8 (C-4), 43.4 (C-14), 42.1 (C-7), 40.8 (C-18), 38.5 (C-8), 37.1 (C-l0), 33.5 (C-21), 32.0 (C-29), 31.7 (C-22), 30.0 (C-20), 27.4 (C-15), 24.9 (C-27), 23.2 (C-11), 22.6 (C-30), 22.5 (C-16), 17.6 (C-25), 17.4 (C-26), 13.8 (C-24).

# Table S1: Physicochemical Properties of 24-deoxysericoside (TM32, TM34-TM37) and the reference drug doxorubicin.

| **Molecule** | **MW** | **#Heavy atoms** | **#Aromatic heavy atoms** | **Fraction Csp3** | **#Rotatable bonds** | **#H-bond acceptors** | **#H-bond donors** | **MR** | **TPSA** |
| --- | --- | --- | --- | --- | --- | --- | --- | --- | --- |
| TM32 | 504.7 | 36 | 0 | 0.9 | 2 | 6 | 5 | 140.14 | 118.22 |
| TM34 | 504.7 | 36 | 0 | 0.9 | 2 | 6 | 5 | 140.14 | 118.22 |
| TM35 | 666.84 | 47 | 0 | 0.92 | 5 | 11 | 8 | 172.11 | 197.37 |
| TM36 | 650.84 | 46 | 0 | 0.92 | 4 | 10 | 7 | 170.95 | 177.14 |
| TM37 | 666.84 | 47 | 0 | 0.92 | 5 | 11 | 8 | 172.11 | 197.37 |
| doxorubicin | 543.52 | 39 | 12 | 0.44 | 5 | 12 | 6 | 132.66 | 206.07 |

# Table S2: Lipophilicity of 24-deoxysericoside (TM32, TM34-TM37) and the reference drug doxorubicin.

| **Molecule** | **iLOGP** | **XLOGP3** | **WLOGP** | **M_LOGP_** | **Silicos-IT Log P** | **Consensus Log P** |
| --- | --- | --- | --- | --- | --- | --- |
| TM32 | -0.46 | 4.5 | 4.15 | 3.33 | 3.46 | 3 |
| TM34 | 2.97 | 4.5 | 4.15 | 3.33 | 3.46 | 3.68 |
| TM35 | 3.38 | 2.69 | 1.41 | 0.54 | 1.41 | 1.88 |
| TM36 | 3.73 | 3.36 | 2.43 | 1.3 | 2.01 | 2.57 |
| TM37 | 4.1 | 2.69 | 1.41 | 0.54 | 1.41 | 2.03 |
| doxorubicin | 2.58 | 1.27 | -0.32 | -2.1 | 1.17 | 0.52 |

# Table S3: Water Solubility of 24-deoxysericoside (TM32, TM34-TM37) and the reference drug doxorubicin.

| **Molecule** | **E_SOL_ Log S** | **E_SOL_ Class** | **Ali Log S** | **Ali Class** | **Silicos-IT LogSw** | **Silicos-IT class** |
| --- | --- | --- | --- | --- | --- | --- |
| TM32 | -5.67 | Moderately soluble | -6.7 | Poorly soluble | -3.9 | Soluble |
| TM34 | -5.67 | Moderately soluble | -6.7 | Poorly soluble | -3.9 | Soluble |
| TM35 | -5.34 | Moderately soluble | -6.49 | Poorly soluble | -2.09 | Soluble |
| TM36 | -5.73 | Moderately soluble | -6.76 | Poorly soluble | -2.67 | Soluble |
| TM37 | -5.34 | Moderately soluble | -6.49 | Poorly soluble | -2.09 | Soluble |
| doxorubicin | -3.91 | Soluble | -5.2 | Moderately soluble | -3.46 | Soluble |

# Table S4: Pharmacokinetics properties of 24-deoxysericoside (TM32, TM34-TM37) and the reference drug doxorubicin.

| **Molecule** | **GI absorption** | **BBB permeant** | **Pgp substrate** | **CYP1A2 inhibitor** | **CYP2C19 inhibitor** | **CYP2C9 inhibitor** | **CYP2D6 inhibitor** | **CYP3A4 inhibitor** | **log Kp (cm/s)** |
| --- | --- | --- | --- | --- | --- | --- | --- | --- | --- |
| TM32 | High | No | Yes | No | No | No | No | No | -6.18 |
| TM34 | High | No | Yes | No | No | No | No | No | -6.18 |
| TM35 | Low | No | Yes | No | No | No | No | No | -8.46 |
| TM36 | Low | No | Yes | No | No | No | No | No | -7.88 |
| TM37 | Low | No | Yes | No | No | No | No | No | -8.46 |
| doxorubicin | Low | No | Yes | No | No | No | No | No | -8.71 |

# Table S5: Druglikeness Properties of 24-deoxysericoside (TM32, TM34-TM37) and the reference drug doxorubicin.

| **Molecule** | **Lipinski #violations** | **Ghose #violations** | **Veber #violations** | **Egan #violations** | **Muegge #violations** | **Bioavailability Score** |
| --- | --- | --- | --- | --- | --- | --- |
| TM32 | 1 | 3 | 0 | 0 | 0 | 0.56 |
| TM34 | 1 | 3 | 0 | 0 | 0 | 0.56 |
| TM35 | 3 | 3 | 1 | 1 | 4 | 0.17 |
| TM36 | 2 | 3 | 1 | 1 | 3 | 0.17 |
| TM37 | 3 | 3 | 1 | 1 | 4 | 0.17 |
| doxorubicin | 3 | 2 | 1 | 1 | 3 | 0.17 |

# Table S6: Medicinal Properties of 24-deoxysericoside (TM32, TM34-TM37) and the reference drug doxorubicin.

| **Molecule** | **PAINS #alerts** | **Brenk #alerts** | **Leadlikeness #violations** | **Synthetic Accessibility** |
| --- | --- | --- | --- | --- |
| TM32 | 0 | 1 | 2 | 6.66 |
| TM34 | 0 | 1 | 2 | 6.68 |
| TM35 | 0 | 1 | 1 | 7.97 |
| TM36 | 0 | 1 | 1 | 7.89 |
| TM37 | 0 | 1 | 1 | 7.94 |
| doxorubicin | 1 | 1 | 1 | 5.81 |
